# Fatal CMV-Infection after Autologous Stem Cell Transplantation in Refractory Systemic Lupus Erythematosus

**DOI:** 10.1155/2012/465089

**Published:** 2012-03-29

**Authors:** László Váróczy, Emese Kiss, Tünde Tarr, Margit Zeher, Gyula Szegedi, Árpád Illés

**Affiliations:** ^1^3rd Department of Medicine, Institute for Medicine, Medical and Health Science Center, University of Debrecen, Móricz Zs. krt. 22, Debrecen 4032, Hungary; ^2^Division of Clinical Immunology and Rheumatology, National Institute for Rheumatology and Physiotherapy, Frankel Leó Strasse 25-29, Budapest H-1023, Hungary

## Abstract

High-dose chemotherapy followed by autologous stem cell transplantation can be a rescue for patients with severe refractory systemic lupus erythematosus (SLE). However, the procedure might have fatal complications including infections and bleeding. We report on a young female patient with SLE whose disease started in her early childhood. After many years, severe renal, neurological, and bone marrow involvement developed that did not respond to conventional therapy. She was selected for autologous stem cell transplantation. A successful peripheral stem cell apheresis was performed in March 2006. The nonselected graft was reinfused in August 2006 after a conditioning chemotherapy containing high-dose cyclophosphamide and antithymocyte globulin. Engraftment was detected within 11 days. On the 38th posttransplant day, severe cytomegalovirus (CMV) infection developed that included pneumonitis, hepatitis, and pancytopenia. The patient died in a week due to multiorgan failure. With her case, we want to call the attention to this rare, but lethal complication of the autologous transplantation.

## 1. Introduction

Systemic lupus erythematosus (SLE) is an autoimmune disorder of multifactorial origin. It is a heterogenous disease ranging from relatively mild condition to severe life-threatening complications involving major organs, such as the kidney, brain, lung, or the bone marrow. Lupus nephritis is the most common major organ manifestation [1]. During the last decades, SLE-related mortality was reduced because of the implementation of more aggressive immunosuppressive medical therapies such as monthly intravenous pulse cyclophosphamide or mycophenolate mofetil [2]. Hopefully, the introduction of biological therapies such as anti-CD20 or anti-CD22 monoclonal antibodies will further improve the treatment results [3]. Despite these advances, there are still some patients whose disease seems to be refractory to any conventional or novel therapeutic modalities. For such patients, high-dose chemotherapy followed or not by autologous stem cell support might be a rescue treatment option. However, autologous hemopoietic stem cell transplantation (AHSCT) has high costs as well as increased risk for fatal complications, so it can be administered only to selected patients [4, 5]. The criteria of patients' selection for AHSCT are shown in Table 1 [6]. 

In the following, we report on a young female patient with refractory SLE who was selected for AHSCT among the first cases in Hungary, but died in the peritransplant period due to a fatal cytomegalovirus infection.

## 2. Case Report

**Figure 1:**
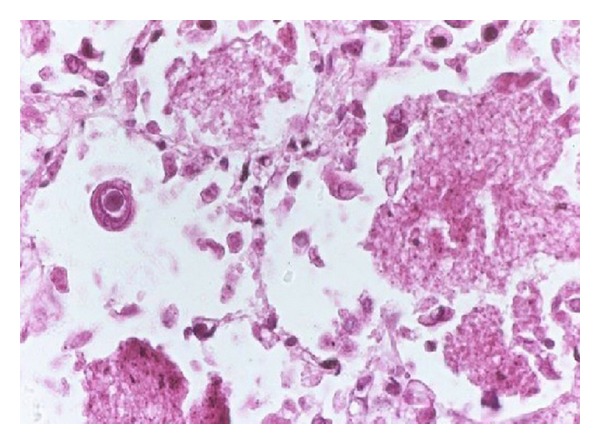
CMV pneumonitis in the patient—autopsy finding (hematoxylin-eosine, 100x magnification).

R. S. was born in 1978 and was diagnosed with systemic lupus erythematosus at the age of eight years. Arthritis, epilepsy, and pericarditis were the first manifestations of her disease. Low-dose prednisolone and azathioprine were administered. In 1995, her neurological complications worsened as frequent epileptic seizures and choreiform dysmotility developed. Tiapride was added to her therapy. In 1997, significant proteinuria (>1 g/day) was diagnosed that indicated kidney biopsy. Histological examination revealed focal segmental glomerulosclerosis (WHO stage III lupus nephritis). Pulse cyclophosphamide treatment was administered for two years, resulting improvement in the renal protein secretion. In 2001, frequent epileptic seizures indicated the introduction of carbamazepine therapy. The patient admitted to our department in 2004 at first. Nonerosive arthritis and fingertip vasculitis were her main complaints. In her laboratory results, we found mild leukopenia (3200/*μ*L), thrombocytopenia (130.000/*μ*L), antinuclear factor positivity, and high titer of antidouble-strand DNA antibody (280 IU/mL). The protein secretion was 1450 mg/day in her urine sample. Cyclosporin A at the dose of 3 mg/bwkg was added to the therapy. In January 2005, cyclosporine A therapy was discontinued due to worsening creatinine clearance and glomerular filtration rate. Pulse cyclophosphamide therapy was restarted at 600 mg/month dose, and it was administered for six months. In December 2005, the activity of her disease increased again: arthritis, leukopenia, thrombocytopenia, and >2 g/day proteinuria were detected. As a severe refractory SLE case, the patient was informed about the opportunity of autologous stem cell transplantation that she accepted. In March 2006, stem cell mobilization was performed. Cyclophosphamide at 2 g/m^2^ dosage was administered followed by mesna support. G-CSF (filgrastim at 10 *μ*g/kg) was added on day 8 when leukocyte count was <2000/*μ*L. On day 10, leukocyte count began to increase, and on day 11, the CD34+ stem cell count was 46/*μ*L. Successful stem cell apheresis was performed as 5.7 × 10^6^/kg CD34+ cells were harvested and frozen without selection. Before the AHSCT, bacterial and viral infections were carefully excluded with appropriate examinations and laboratory tests, including sputum, urine, and stool culturing. Both cytomegalovirus (CMV) and Epstein-Barr virus (EBV) serology tests showed IgG positivity. The patient was admitted to the stem cell transplantation unit on August 1, 2006. The conditioning regimen consisted of high-dose cyclophosphamide (200 mg/kg in 4 fractions) and rabbit antithymocyte globulin (ATG) at 15 mg/kg. ATG was given on days −3 and −2 as well as on day +2 for *in vivo* purging. Stem cells were reinfused on August 7. In the first posttransplant days, gastroenteritis and mild bleeding complication appeared, but both of them were resolved. During the neutropenic days, combined antimicrobial prophylaxis including ciprofloxacin, trimethoprim/sulfamethoxazole, fluconazole, and acyclovir was administered. Engraftment was detected on day +11; however, thrombocytopenia lasted for 20 days. The patient could go home on day +25 in good condition. The first posttransplant checkup was on day +32 when a positive cytomegalovirus antigen test was detected. The patient had no complaints, and all her blood cell counts were normal. Oral valganciclovir therapy was started. On day +38, she appeared with high fever. Febrile neutropenia could be diagnosed, and her absolute neutrophil count (ANC) decreased to 200/*μ*L. She was readmitted to the stem cell transplantation unit and broad-spectrum antibiotic (meropenem), and intravenous ganciclovir therapy was given to her. Within 24 hours, severe acute respiratory insufficiency developed. The chest X-ray picture showed respiratory distress syndrome (ARDS). Elevated levels of liver enzymes (AST, ALT, and alkaline phosphatase) could be also detected. Respiration therapy was started, and high-dose intravenous immunoglobulin was also added to the supportation. Unfortunately, there was no improvement in the patient's state, and she died on the 46th posttransplant day due to multiorgan failure. The autopsy confirmed the presence of giant-cell pneumonitis that is typical for severe CMV infection (Figure 1).

## 3. Discussion

High-dose chemotherapy followed by autologous stem cell support can be a promising therapeutic approach for severe, refractory autoimmune disorders with life-threatening complication. In this procedure, immunoablative chemotherapy destroys the autoimmune clones, while the reinfused stem cells repopulate the bone marrow and rebuild a hopefully well-functioning immune system. Since 1996, approximately 130–140 SLE patients have been enrolled in early studies of autologous stem cell transplantation worldwide. The largest number of cases was published by the EULAR/EBMT database and the Immunotherapy Center of the Northwestern University in Chicago. Most patients had progressive disease with a predominant kidney, lung, bone marrow, or nervous system involvement and were nonresponders for conventional immune-modulator treatment. The stem cell source was rather peripheral blood than bone marrow, and the most common method of stem cell mobilization was a combination of cyclophosphamide (2–4 g/m^2^) and granulocyte colony-stimulating factor (G-CSF). CD34+ selection was used for further T-cell depletion in about half of the cases. The conditioning regimens included high-dose cyclophosphamide (200 mg/kg), antithymocyte globulin at various doses, or total body irradiation (TBI) in some cases [6–8]. Four to eight percent of the patients died because of transplant-related complications (infections, bleeding) within the first 100 days. ASCT was beneficial, and a significant clinical improvement was observed by most of the patients at the early posttransplant period. Mean before transplant SLEDAI scores fell from 33.2 before transplant to a mean of 2.6–3.8 after transplant. Antinuclear antibody became negative in 80% of evaluable patients, and similar normalization was observed in anti-dsDNA antibodies. However, these abnormalities reappeared in about one-quarter of the patients subsequently. Maintenance steroid therapy could be discontinued for good and all only in 35–40% of the cases, but sustained improvement could be observed by more than half of the patients after a median followup of 3 years. Referring the data from the Northwestern University, the probability of overall and disease-free survivals was 85% and 50% at 5 years, respectively. Pilot studies on SLE patients underlined the fact that the CD34+ AHSCT is a very beneficial immune cell therapy in the disease, yet further, more extensive, randomized studies are needed to elucidate the role of this intervention in the therapy for patients with SLE [9]. 

Cytomegalovirus infection is a major complication after allogenic stem cell or solid organ transplantation. The most frequent manifestations of CMV disease are interstitial pneumonia, gastroenteritis, and hepatitis. Rare manifestations are retinitis and encephalitis. Active CMV infection might be accompanied by myelosuppression and fever [10]. Virus-specific antigens are produced in tissue culture long before cytopathic effects are detectable and can be identified with specific monoclonal antibodies [11]. Quantitative PCR assay is another effective way to detect cytomegaloviruses in the whole blood samples of the patients [12]. In the prevention of cytomegalovirus disease, the use of acyclovir, valacyclovir, or ganciclovir all seemed to be effective [10].

The risk for CMV disease after autologous stem cell transplantation is generally low. It can be explained with the fact that the reconstitution of CMV-specific T cells occurs within a short while after HSCT and associates with a short, mostly self-limited period of active CMV replication not requiring antiviral therapy on the majority of the patients [13]. Some authors do not even recommend the screening of CMV antigenemia after autologous stem cell support. However, certain patient groups can have an increased risk. Holmberg et al. reported a 22.6% incidence of CMV disease among autotransplanted patients who received CD34+ selected grafts [14]. In our case, several independent risk factors could have been in the background. The young female patient had been on constant immunosuppressive therapy since her early childhood. The conditioning regimen contained antithymocyte globulin, causing long-lasting T-lymphopenia. Last but not least, she received several packages of red blood cell and platelet transfusions. However, she was CMV seropositive before the transplant procedure and leukocyte-depleted blood products were administered, it cannot be excluded that the transfusions also contributed to the development of lethal CMV infection.

Learning from the unsuccessful lesson of this case, we strongly recommend to perform the CMV assays after AHSCT of autoimmune patients. In case of a positive result, antiviral prophylaxis and thorough observation may be needed.

## Figures and Tables

**Table 1:** Inclusion and exclusion criteria for autologous stem-cell transplantation in systemic lupus erythematosus.

Inclusion criteria	Exclusion criteria
(1) The diagnostic criteria for SLE are fulfilled based on the ACR guidelines (2) Type III or IV glomerulonephritis based on the WHO classification cannot be controlled by short-term treatment with cyclophosphamide (3) Lung and/or cardiac parenchymal destruction due to histologically proven vasculitis, if not improving to IV cyclophosphamide (4) Encephalitis or myelitis transversa, if not improving to IV cyclophosphamide (5) Severe refractory autoimmune cytopenia, if refractory to standard-dose treatment (6) Catastrophic antiphospholipid syndrome	(1) Absolute neutrophil counts <2000/*μ*L (2) Platelet counts <120000/*μ*L (3) Age over 70 years (4) Serum creatinine level is over 3,0 mg/dL (5) Ejection fraction is below 45% by echocardiography, malignant arrhythmia, and NYHA stage IV cardiac failure (6) Accompanying malignant disease (7) Infection (8) Respiratory failure (9) Deterioration of liver functions

Abbreviations: ACR: American College of Rheumatology, WHO: World Health Organization, NYHA: New York Heart Association.
